# Mitochondrial targeted antioxidants, mitoquinone and SKQ1, not vitamin C, mitigate doxorubicin-induced damage in H9c2 myoblast: pretreatment vs. co-treatment

**DOI:** 10.1186/s40360-021-00518-6

**Published:** 2021-09-16

**Authors:** Brian Sacks, Halil Onal, Rose Martorana, Amogh Sehgal, Amanda Harvey, Catherine Wastella, Hafsa Ahmad, Erin Ross, Adona Pjetergjoka, Sachin Prasad, Robert Barsotti, Lindon H. Young, Qian Chen

**Affiliations:** Department of Bio-Medical Sciences, Philadelphia College of Osteopathic Medicine, 4170 City Avenue, Philadelphia, PA 19131 USA

**Keywords:** Doxorubicin, Cardiotoxicity, Mitochondrial-targeted antioxidants, Vitamin C, Oxidative stress

## Abstract

**Background:**

Preconditioning of the heart ameliorates doxorubicin (Dox)-induced cardiotoxicity. We tested whether pretreating cardiomyocytes by mitochondrial-targeted antioxidants, mitoquinone (MitoQ) or SKQ1, would provide better protection against Dox than co-treatment.

**Methods:**

We investigated the dose-response relationship of MitoQ, SKQ1, and vitamin C on Dox-induced damage on H9c2 cardiomyoblasts when drugs were given concurrently with Dox (e.g., co-treatment) or 24 h prior to Dox (e.g., pretreatment). Moreover, their effects on intracellular and mitochondrial oxidative stress were evaluated by 2,7-dichlorofluorescin diacetate and MitoSOX, respectively.

**Results:**

Dox (0.5–50 μM, *n* = 6) dose-dependently reduced cell viability. By contrast, co-treatment of MitoQ (0.05–10 μM, *n* = 6) and SKQ1 (0.05–10 μM, *n* = 6), but not vitamin C (1–2000 μM, *n* = 3), significantly improved cell viability only at intermediate doses (0.5–1 μM). MitoQ (1 μM) and SKQ1 (1 μM) significantly increased cell viability to 1.79 ± 0.12 and 1.59 ± 0.08 relative to Dox alone, respectively (both *p* < 0.05). Interestingly, when given as pretreatment, only higher doses of MitoQ (2.5 μM, *n* = 9) and SKQ1 (5 μM, *n* = 7) showed maximal protection and improved cell viability to 2.19 ± 0.13 and 1.65 ± 0.07 relative to Dox alone, respectively (both *p* < 0.01), which was better than that of co-treatment. Moreover, the protective effects were attributed to the significant reduction in Dox-induced intracellular and mitochondrial oxidative stress.

**Conclusion:**

The data suggest that MitoQ and SKQ1, but not vitamin C, mitigated DOX-induced damage. Moreover, MitoQ pretreatment showed significantly higher cardioprotection than its co-treatment and SKQ1, which may be due to its better antioxidant effects.

## Introduction

Doxorubicin (Dox) is a widely used anti-cancer drug due to its ability to generate free radicals and interfere with DNA replication on cancer cells. However, Dox also induces severe cardiotoxicity, such as cardiomyopathy and associated heart failure [1, 2]. Dexrazoxane is a drug used to reduce Dox-induced cardiotoxicity possibly by chelating iron and inhibiting Dox-topoisomerase II (TOP II) interaction. On the other hand, dexrazoxane can cause hematological toxicity, gastrointestinal discomfort, and even increase the risk of developing a second primary malignancy, which limit its use in women with advanced or metastatic breast cancer [3, 4]. Therefore, it is integral to identify new strategies to protect the heart against Dox during chemotherapy.

Dox induces cardiac cell damage and/or apoptosis through the same mechanisms as its ability to kill cancer cells. It intercalates between DNA strands as well as inhibits the function of TOP II, leading to blockage of DNA replication and transcription and breakage of DNA double strand [3, 5]. This is somewhat selective for cancer cells because they are replicating more than most normal cells. Dox also induces cardiotoxicity by accumulating within the mitochondria and increasing reactive oxygen species (ROS) production [6]. Dox can additionally lead to a reduction in glutathione peroxidase and catalase levels to decrease endogenous anti-oxidant capacity [7]. Another feature is that Dox can interact with cardiolipin to disrupt proper functioning of electron transport chain in mitochondria [1, 2]. Because cardiac tissue is most abundant in mitochondria compared to other tissues, the heart is the most susceptible to Dox-induced mitochondrial damage. Thus, protection of mitochondria against Dox-induced oxidative stress would be an effective strategy to mitigate cardiotoxicity under Dox treatment.

Vitamin C is a representative common anti-oxidant, which has been widely used, in daily life. Most studies have shown vitamin C can protect the heart against Dox by enhancing antioxidant and anti-inflammation effects and reducing cellular damage and apoptosis in vitro and in vivo studies [5, 8]. However, higher concentrations of vitamin C have been suggested to be pro-oxidant which can damage the cell [9]. Furthermore, vitamin C may not specifically target the mitochondria when compared to mitochondria-targeted antioxidants, such as mitoquinone (MitoQ) and 10-(6′-plastoquinonyl) decyltriphenylphosphonium (SKQ1). MitoQ and SKQ1 are a derivative of ubiquinone or plastoquinone conjugated to decyl-triphenylphosphonium cation (TPP), respectively [10, 11]. TPP is a lipophilic cation, and conjugation allows the compound to concentrate in the mitochondria with ease due to the powerful electrical gradient created between the cationic TPP and drastic negative membrane potential of the mitochondria (eg., − 160 mV to − 180 mV) [10]. Moreover, MitoQ is available over counter for antioxidant supplement use and SKQ1 (Visomitin) is approved to treat dry eye conditions in Russia [10]. It’s known that MitoQ reduces the production of lipid peroxyl radicals and prevents lipid peroxidation [12]. MitoQ is also demonstrated to reduce Dox-induced damage in H9c2 cells and cardiomyopathy in animal studies [13]. By contrast, SKQ1 has been found to form a complex with cardiolipin in order to prevent lipid peroxidation [14]. SKQ1 also shows a four-fold higher decrease in peroxyl radicals than MitoQ in vitro studies [11]. However, the effects of SKQ1 on Dox-induced cardiotoxicity have not been well studied. Thus, it is worthwhile to investigate if SKQ1 would provide the same or better cardioprotection against Dox than vitamin C or MitoQ. Since MitoQ and SKQ1 can also serve as pro-oxidants at higher concentrations [15], it is important to understand their dose-response relationship related to those effects. Recently, two labs have indicated that preconditioning of cardiomyocytes by transient hypoxia/reoxygenation or remote ischemic preconditioning ameliorates Dox-induced cardiotoxicity and preserves mitochondrial functions [16, 17]. Moreover, MitoQ pretreatment is found to upregulate antioxidant genes, attenuate mitochondrial oxidative stress, and prevent mitochondrial DNA depletion under intestinal ischemia/reperfusion conditions [18]. However, there is lack of research demonstrating the effects of mitochondria-targeted antioxidants when given as pretreatment on Dox-induced cardiac cell damage.

In this study, we first determined dose-dependent effects of Dox on the cellular damage in H9c2 cells. We also compared Dox’s effects to hydrogen peroxide (H_2_O_2_), a common oxidative stress inducer. We then compared the effects of vitamin C, MitoQ, or SKQ1 on Dox-induced cell damage when drugs were given concurrently with Dox (e.g., co-treatment) or 24 h prior to adding Dox (e.g., pretreatment). Lastly, we investigated the effects of Dox alone or combined with MitoQ or SKQ1 on intracellular and mitochondrial oxidative stress.

## Methods

### H9c2 myoblast

Rat H9c2 cells (CRL-1446, American Type Culture Collection (ATCC), Manassas, VA) were cultured in 75 cm^2^ flasks and petri dishes using 4.5 g/mL glucose DMEM with 10% fetal bovine serum and 1% penicillin streptomycin solution (Corning, Fisher Scientific, Waltham, MA) at 37 °C and 5% CO_2_ in a humid incubator. Cell media was changed every 2–3 days till cells reached 70–80% confluence for passaging to prevent the loss of differentiation potential according to the ATCC instruction. Meanwhile, some cells were harvested for experiments. Cell density after the collection was counted using 0.3% trypan blue kit (Sigma-Aldrich, St. Louis, MO) and plated 2 × 10^4^ cells per well in a 96-well plate. After seeding, H9c2 cells were incubated for 24 h before treatments were administered. We used H9c2 cells within 5–20 passages for all the experiments because control cells showed similar morphology under light microscopy (200X). Cell responses to all tested compounds were very close across all the passages. All the procedures for maintaining the cell line and conducting experiments were carried out in a cell culture hood with sterile techniques. Cells were checked for contamination, morphology alteration, and confluence by microscopy every day. When cells were contaminated or deformed, all the flasks, media, and cell plates were immediately discarded. The cell incubator was cleaned and disinfected. Then, a new H9c2 cell line was freshly restarted. All experiment groups included a non-treated cell control to observe any physical, chemical, and physiological changes among groups and cell passages. To prevent chemical contamination, different treatments were added to cells in separate columns on a cell plate. Moreover, pipette tips were frequently changed during procedures.

### Experimental groups

#### Dox or H_2_O_2_ induced cell damage

Dox (0.5–50 μM; M.W. =579.98 g/mol, Sigma-Aldrich, St. Louis, MO) or H_2_O_2_ (100–600 μM; M.W. =34.01 g/mol, Sigma-Aldrich, St. Louis, MO) and solvent control (e.g., medium) were applied to cells which were in 1-day-old medium (e.g., after cell seeding for 24 h) to determine cell damage. When cells were pretreated with testing compounds, fresh medium was added after washing out the testing drugs. We have found that fresh medium would reduce the cell damage induced by H_2_O_2_. Therefore, Dox (0.1–50 μM) or H_2_O_2_ (100–600 μM) and solvent control (e.g., medium) were applied to cells with fresh medium to determine if there is any different effects of Dox or H_2_O_2_ on cells with fresh medium when compared to that with 1-day-old medium. Cells were then incubated in Dox or H_2_O_2_ for 24 h before performing biochemical assays.

#### Vitamin C treatments

First, vitamin C (1–2000 μM; M.W. =276.12 g/mol, Sigma-Aldrich, St. Louis, MO) was administered to cells alone to determine if vitamin C itself caused any cell damage. Then, vitamin C (1–2000 μM) was administered with Dox (40 μM) as a co-treatment to evaluate if vitamin C would exert any protection against Dox. Cells were incubated for another 24 h before performing biochemical assays.

#### MitoQ treatments

MitoQ (0.05–10 μM; M.W. =678.8 g/mol, Cayman Chemical, Ann Arbor, Michigan) was applied to cells alone to test if MitoQ itself caused any cell damage. Furthermore, MitoQ was administered with Dox (40 μM) as a co-treatment to evaluate if MitoQ would exert any protection against Dox. We also used MitoQ as a pretreatment by adding MitoQ to cells for 24 h. Then MitoQ was removed and cells were washed with PBS twice, then fresh medium was added prior to administration of Dox (40 μM). Cells were incubated for another 24 h before performing biochemical assays.

#### SKQ1 treatments

Similarly as MitoQ, SKQ1 (0.05–10 μM; M.W. =617.6 g/mol, Cayman Chemical, Ann Arbor, Michigan) was applied to cells alone to test if SKQ1 itself caused any cell damage. Furthermore, SKQ1 was administered as a co-treatment or pretreatment to determine if SKQ1 exerted any cell protection against Dox (40 μM).

### Biochemical assays

#### Cell viability analysis by cell counting kit-8 (CCK-8)

CCK-8 (Dojindo Molecular Technologies, Rockville, MD) was used to evaluate cell viability based on intracellular dehydrogenase activity. Tetrazolium salt is reduced by dehydrogenases in live cells to form formazan, which is soluble in tissue, and the amount of formazan dye generated by dehydrogenases is directly proportional to the number of living cells. After the corresponding treatments described above, the medium was removed, and cells were washed twice with PBS, then 100 μl fresh medium was added. CCK reagent (10 μl) was added to each well and cells were incubated for 4 h to allow the reaction to complete. Absorbance was measured at 450 nm with ELISA plate reader, and the ratio of absorbance in treated vs. non-treated (e.g., medium) control or Dox alone was determined.

#### Cell viability by calcein assay

To further confirm cell damage caused by Dox or H_2_O_2_, calcein assay was used to stain live cells. Calcein is a cell permeable dye that is converted to green fluorescent calcein after acetoxymethyl ester is hydrolyzed by intracellular esterase in live cells. Calcein (Thermo Fisher Scientific, Waltham, MA) was prepared using PBS. After treatment of Dox or H_2_O_2_ for 24 h, culture media was removed. Calcein solution (3 μM) was added to each well and fluorescence intensity was measured at 485 nm/535 nm with Fluoroskan Ascent CF after incubation for 30 min. The ratio of fluorescence intensity in treated vs. non-treated (e.g., medium) control was determined.

#### Intracellular ROS analysis by 2, 7-dichlorofluorescin diacetate (DCFDA)

DCFDA (Thermo Fisher Scientific, Waltham, MA) is a fluorogenic dye that allows quantification of hydroxyl, peroxyl and other ROS activity within the cell. Cell permeable DCFDA is non- fluorescent compound which is first deacylated by cellular esterase inside live cells, and then oxidized by intracellular ROS to DCF, which is a highly fluorescent compound that can be detected by fluorescence spectrometry. First, H9c2 cells were loaded with DCFDA after they were incubated with100 μl of DCFDA (25 μM) for 45 min. Then, DCFDA reagent was removed and 100 μl of DMEM medium was added to each well. Thereafter, cells were treated with Dox (0.1–50 μM) or Dox (40 μM) combined with MitoQ or SKQ1 to determine the effects of Dox alone or co-treatment of drugs (MitoQ or SKQ1) on intracellular ROS. In another set of experiments, DCFDA was added to cells after pretreatment of MitoQ or SKQ1 or vehicle for 24 h. After removing DCFDA, cells were treated with Dox (40 μM) to determine the effects of MitoQ or SKQ1 pretreatment on Dox-induced intracellular ROS. Fluorescence intensity was measured at 485 nm/535 nm by Fluoroskan Ascent CF at 10 min and 24 h after cells were incubated with Dox, The intracellular ROS levels were expressed as the ratio between Dox to non-treated control or between pretreatment to Dox, respectively.

#### Mitochondrial superoxide (SO) analysis by MitoSOX

MitoSOX (Thermo Fisher Scientific, Waltham, MA) is a novel fluorogenic dye used for detection of SO within the mitochondria of live cells. The lipophilic, positively charged TPP moiety within MitoSOX allows the dye to concentrate within the mitochondrial matrix pending on high mitochondrial membrane potential in live cells. The dye is oxidized by SO to produce red fluorescence. First, cells were treated by Dox (0.1–50 μM) alone, Dox (40 μM) co-treated with MitoQ or SKQ1, or Dox (40 μM) following pretreatment of MitoQ or SKQ1. After 24 h, the medium was removed and cells were washed with PBS. Thereafter, 100 μl MitoSOX (5 μM) reagent was added to each well and incubated for 15 min. Fluorescence intensity was measured after changing the incubation solution to medium at 531 nm/ 593 nm with Fluoroskan Ascent CF. The mitochondrial SO levels were expressed as the ratio between Dox to non-treated control or between pretreatment to Dox, respectively.

### Statistical analysis

All experiments were performed in triplicate and repeated at least three times. The data was expressed by mean ± SE. The data were analyzed using ANOVA followed by Student Newman Keuls and *p* value of < 0.05 was considered as statistical significance.

## Results

### Dox dose-dependently decreased cell viability similarly as H_2_O_2_, but culture medium did not affect its cytotoxicity

**Fig. 1:**
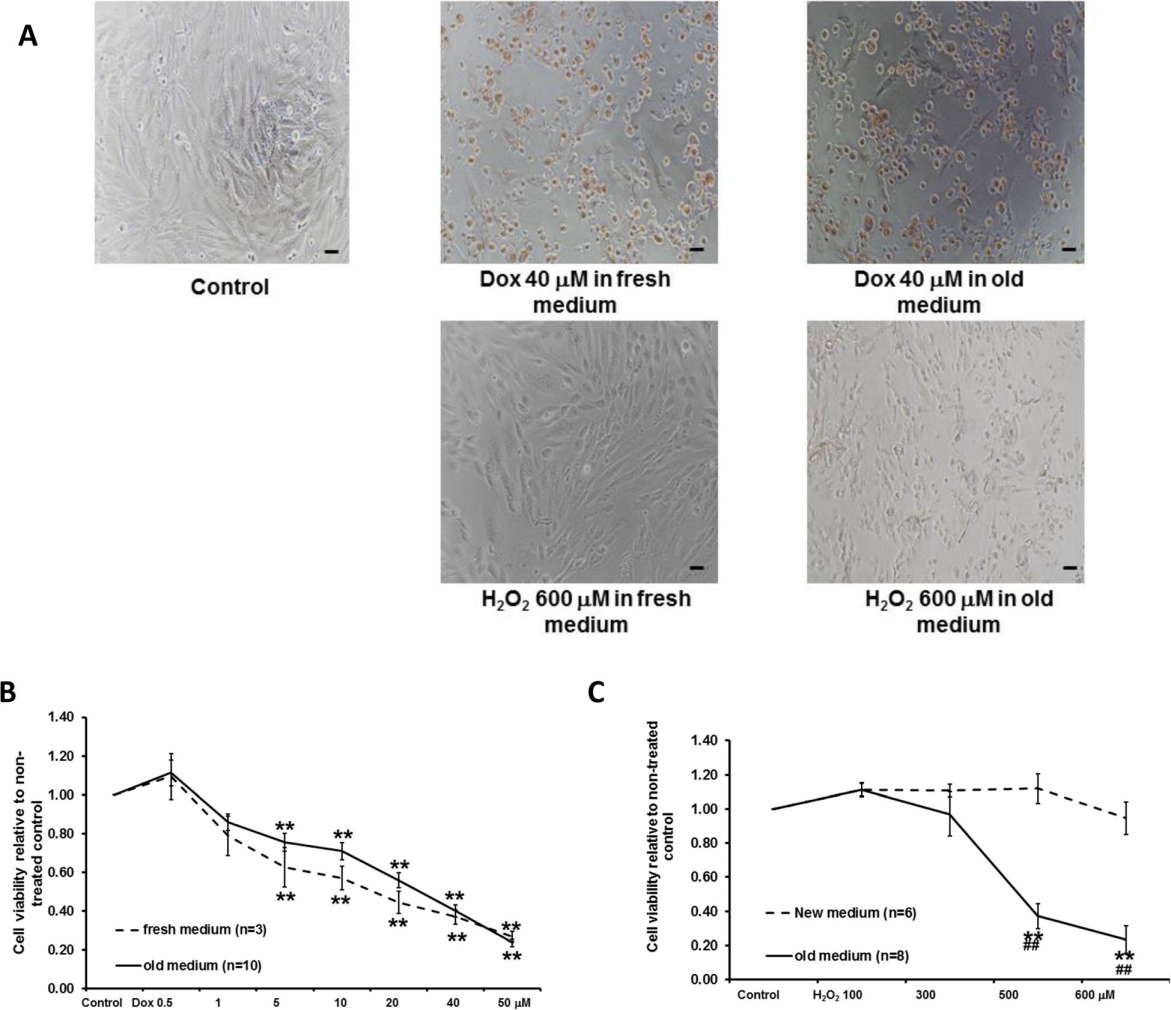
Effects of Dox or H_2_O_2_ on H9c2 cells when Dox or H_2_O_2_ were added to a fresh or 1-day-old medium. The representative pictures of cells (100 X) after Dox or H_2_O_2_ incubation are shown in **A** (scale bar: 50 μm). Dose-dependent effects of Dox or H_2_O_2_ on cell viability are shown in **B** and **C**, respectively. Unlike H_2_O_2_, the effects of Dox were not significantly different when Dox was applied to fresh vs old medium. **p < 0.01 vs non-treated control; ##*p* < 0.01 vs fresh medium

The effects of Dox (0.5–50 μM) or H_2_O_2_ (100–600 μM) on H9c2 cell viability under fresh and 1-day old medium is shown in Fig. 1A-C. Compared to non-treated control cells, treatment with Dox doses ranging from 5 μM to 50 μM significantly reduced cell viability (see Fig. 1A-B). The reduction was dose-dependent. However, the dose-response of Dox on cell viability was not significantly different when Dox was directly added to 1-day-old medium (*n* = 10) or fresh medium (*n* = 3). Dox (40 μM) significantly reduced cell viability to 0.40 ± 0.03 or 0.37 ± 0.04 under old or fresh medium, respectively (both *p* < 0.05 compared to non-treated control). Similarly, H_2_O_2_ (100–600 μM) reduced cell viability in a dose-dependent manner (see Fig. 1A and C). However, the cytotoxicity induced by H_2_O_2_ (500 μM and 600 μM) was significantly lower when H_2_O_2_ was added to 1-day-old medium (*n* = 8) compared to those in fresh medium (*n* = 6). After incubation of 600 μM H_2_O_2_ for 24 h, cell viability relative to non-treated control in 1-day-old medium (0.24 ± 0.08, *n* = 8, *p* < 0.01) was significantly lower than in fresh medium (0.95 ± 0.10, *n* = 6).

**Fig. 2:**
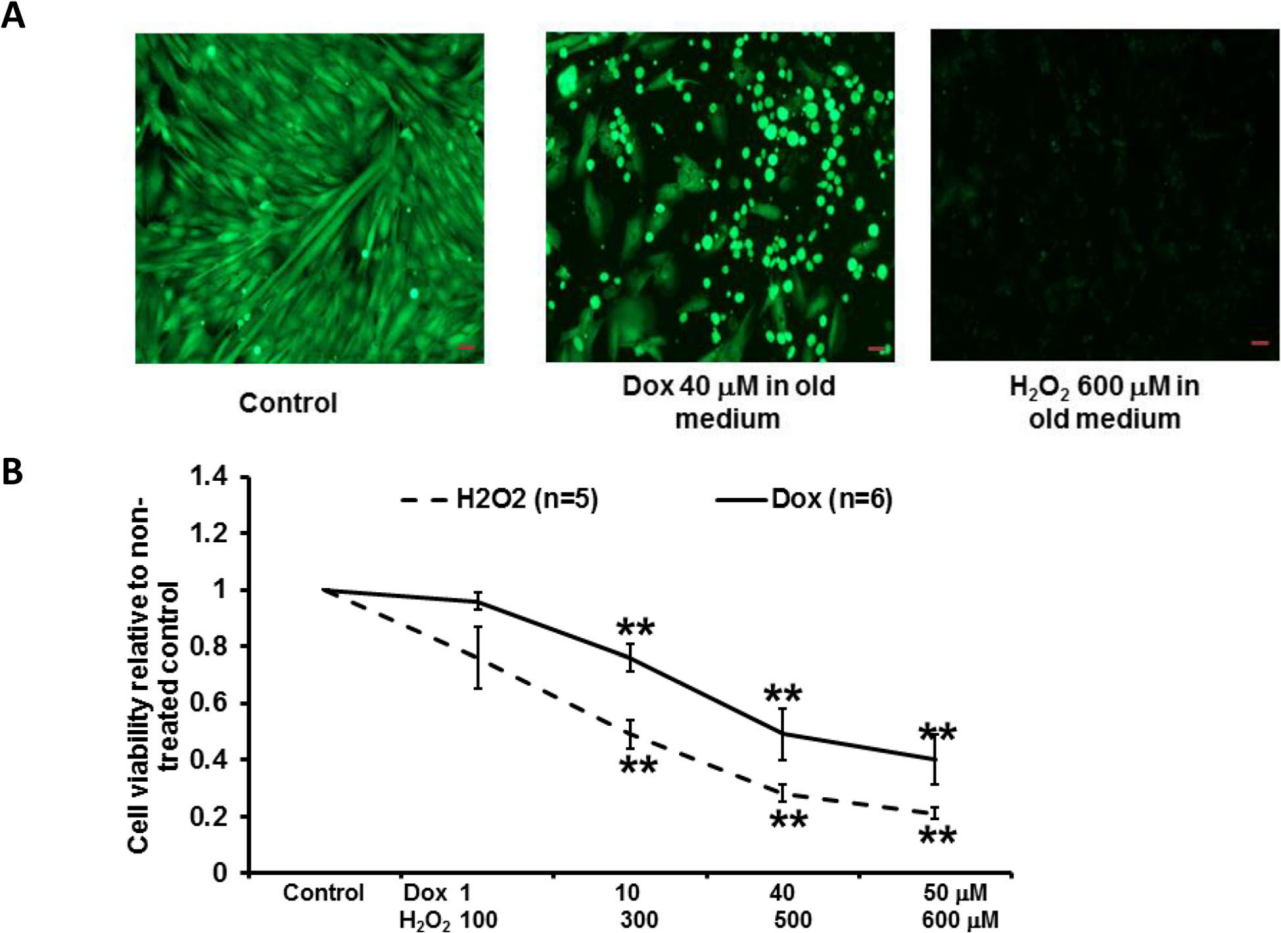
Effects of Dox or H_2_O_2_ on H9c2 cell viability by a calcein staining assay when Dox or H_2_O_2_ were added to 1-day-old medium. The representative pictures of stained cells (100 X) after Dox or H_2_O_2_ incubation are shown in **A** (scale bar: 50 μm). Dose-dependent effects of Dox or H_2_O_2_ on cell viability are shown in **B**. Dox or H_2_O_2_ dose-dependently reduced cell viability. **p < 0.01 vs non-treated control

We further confirmed the cell damage by Dox and H_2_O_2_ in 1-day old medium by calcein staining (see Fig. 2A-B). We found that cells after incubation of Dox or H_2_O_2_ exhibited much less green staining when compared to non-treated control (see Fig. 2A). Also, Dox (*n* = 6) and H_2_O_2_ (*n* = 5) reduced cell viability in a dose-dependent manner (see Fig. 2B), and the effects were similar as those evaluated by CCK assay. Dox (40 μM) and H_2_O_2_ (600 μM) significantly reduced cell viability to 0.49 ± 0.09 and 0.21 ± 0.02, respectively, when compared to the non-treated control (both *p* < 0.01). In the following experiments, Dox (40 μM) was used with antioxidants to determine if vitamin C, SKQ1, or MitoQ would protect cells against Dox-induced cell damage.

### Antioxidants alone did not induce any cell damage

**Fig. 3:**
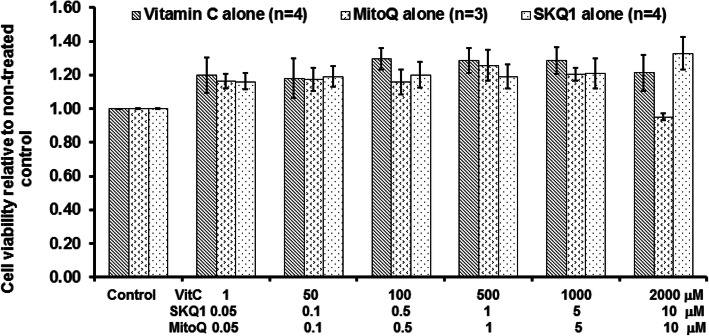
The effects of vitamin C, MitoQ, or SKQ1 on cell viability. There was no significant change in cell viability by all the three antioxidants

The effects of antioxidants alone on cell viability are illustrated in Fig. 3. All three antioxidants (*n* = 3–4) slightly increased cell viability which was determined by measuring live cell dehydrogenase activity. Moreover, higher dose MitoQ (10 μM) showed a slight reduction in cell viability. However, there was no significant difference when compared to non-treated control or each other.

### Mitochondrial targeted antioxidants, but not vitamin C, protected H9c2 cells against Dox when given as co-treatment

**Fig. 4:**
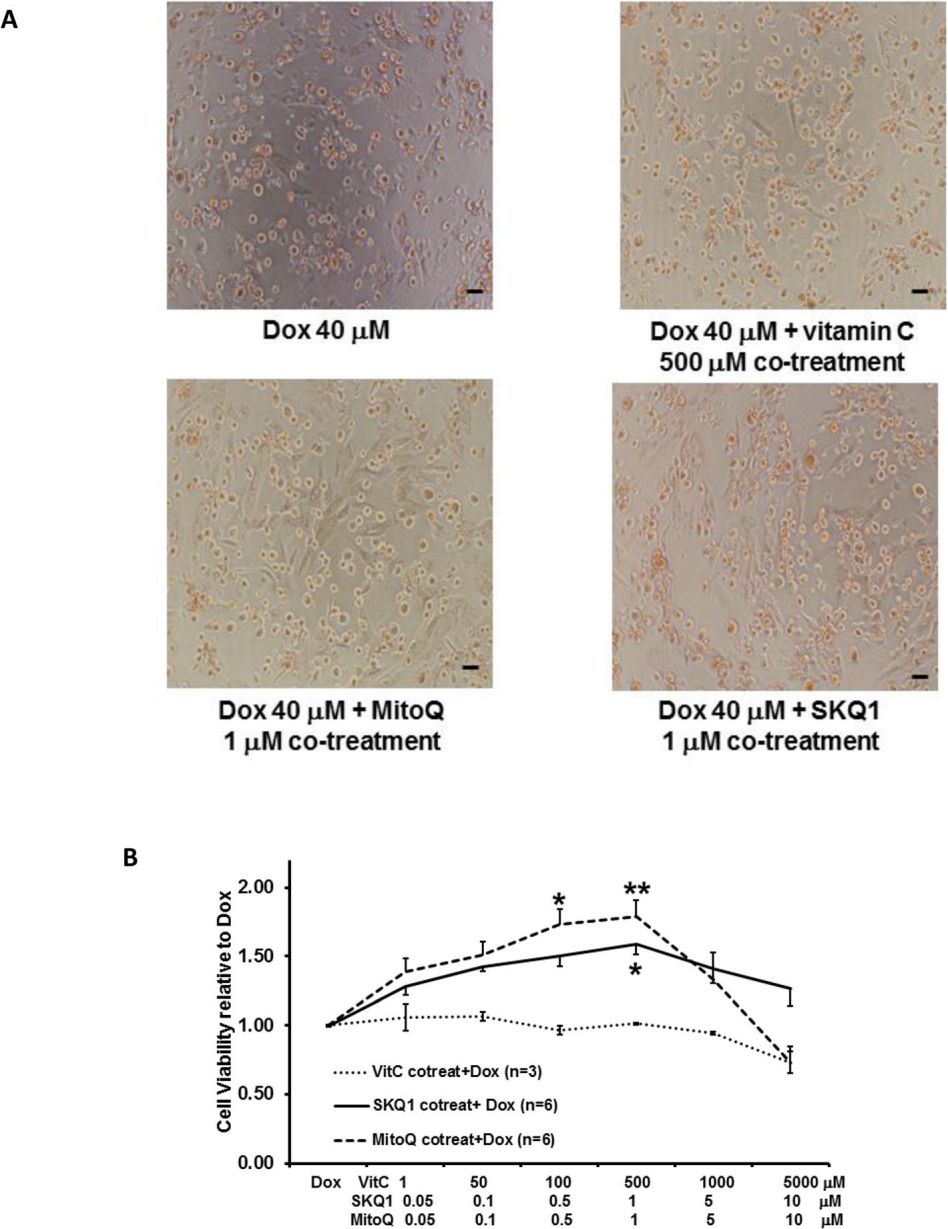
The effects of vitamin C, MitoQ, or SKQ1 on cell viability against Dox when given as co-treatment. The representative pictures of cells (100 X) after co-treatment are shown in **A** (scale bar: 50 μm). The effects of different antioxidants on Dox-induced cell damage are illustrated in **B**. MitoQ and SKQ1, but not vitamin C, significantly increased cell viability in the presence of Dox. *p < 0.05; **p < 0.01 vs Dox

The effects of the different combinations of antioxidants with 40 μM Dox on cell viability is shown in Fig. 4A-B. Co-treatment of MitoQ or SKQ1 with Dox showed more live cells than those in Dox alone or vitamin C treatment (see Fig. 4A). Also, we found that vitamin C (1–2000 μM, *n* = 3) did not show any increase in cell viability at any given dose. By contrast, SKQ1 (0.05–10 μM, *n* = 6) and MitoQ (0.05–10 μM, *n* = 6) increased cell viability in a dose-dependent manner (see Fig. 4A-B). MitoQ showed slightly better protective effects than SKQ1, but did not show any significant difference between them. MitoQ (1 μM) and SKQ1 (1 μM) significantly increased cell viability to 1.79 ± 0.12 and 1.59 ± 0.08, respectively, when compared to Dox (both *p* < 0.05). Interestingly, we found the protective effects exhibited by SKQ1 and MitoQ were either reduced or lost when the doses were higher than 1 μM, respectively.

### MitoQ and SKQ1 provided better protection against Dox when given as pretreatment than co-treatment

**Fig. 5:**
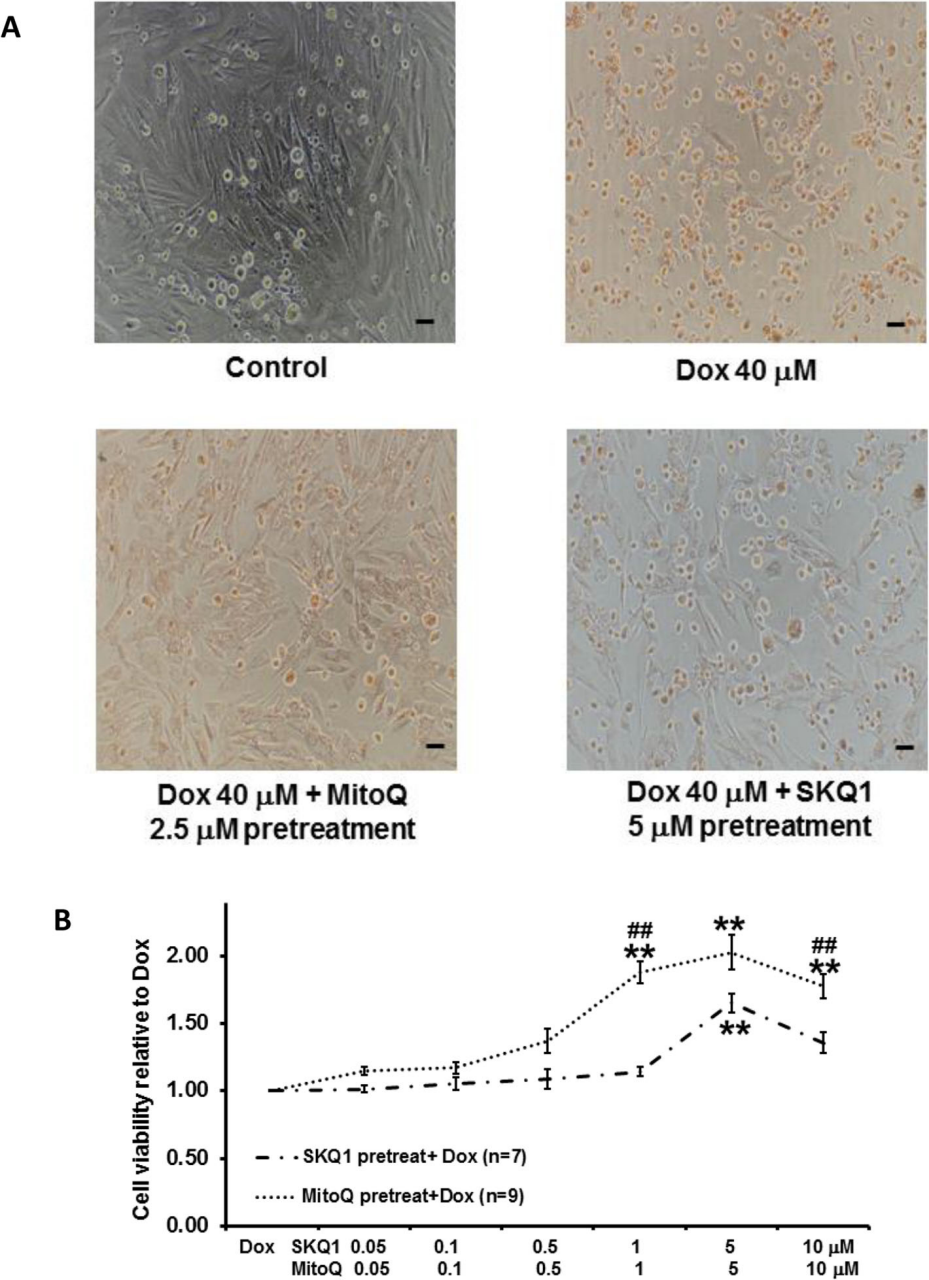
The effects of pretreatment of MitoQ or SKQ1 on Dox-induced cell viability reduction. The representative pictures of cells (100 X) after pretreatment are shown in **A** (scale bar: 50 μm). The effects of MitoQ and SKQ1 pretreatment on Dox-induced cell damage are illustrated in **B**. Higher doses of MitoQ or SKQ1 significantly improved cell viability when given as pretreatment. Moreover, MitoQ exerted significantly higher protection than SKQ1. *p < 0.05; **p < 0.01 vs Dox; ##*p* < 0.01 vs SKQ1

The effects of pretreating cells by SKQ1 and MitoQ on Dox-induced cell damage are shown in Fig. 5A-B. Pretreatment of MitoQ or SKQ1 showed more live cells than those in Dox alone (see Fig. 4A). Moreover, we found that SKQ1 (0.05–10 μM, *n* = 7) and MitoQ (0.05–10 μM, *n* = 9) increased cell viability in a dose-dependent manner. MitoQ (5 μM) and SKQ1 (5 μM) exerted the maximal effect and showed a significant increase in cell viability (2.03 ± 0.13 and 1.65 ± 0.07, respectively) when compared to Dox (40 μM, both *p* < 0.05) (see Fig. 5B). Furthermore, MitoQ showed significantly better protective effects than SKQ1 at 1 μM and 10 μM (see Fig. 5B).

**Fig. 6:**
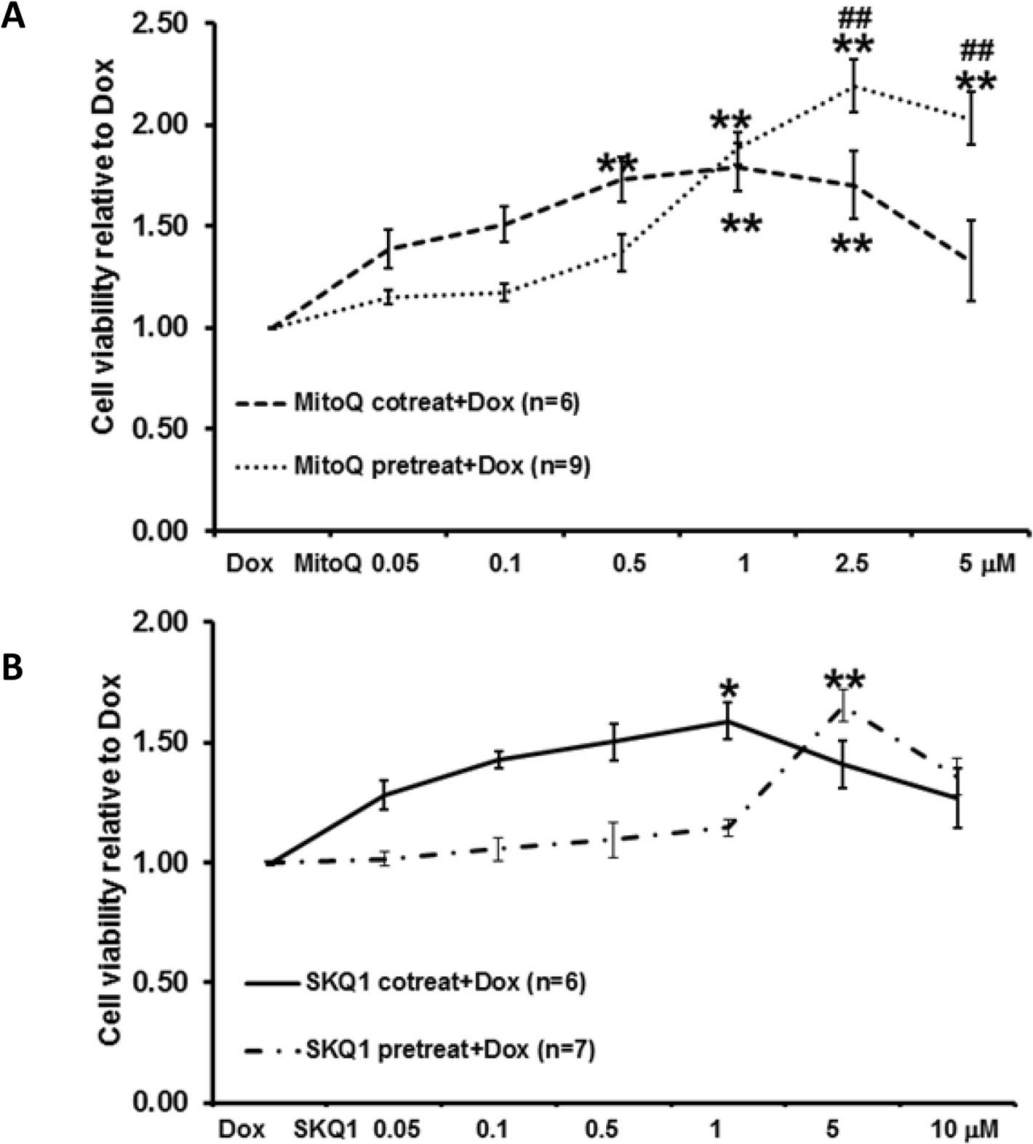
The comparison between pretreatment and co-treatment of MitoQ (**A**) and SKQ1 (**B**) on Dox-induced cell damage. Pretreatment and co-treatment of MitoQ and SKQ1 significantly protected cells against Dox by demonstrating higher cell viability. Moreover, pretreatment of MitoQ, not SKQ1, exerted significantly higher protection than co-treatment at higher doses. *p < 0.05; **p < 0.01 vs Dox; ##*p* < 0.01 vs MitoQ co-treatment

Furthermore, we found that intermediate doses of MitoQ (0.05–0.5 μM) exerted better protection when given as co-treatment than pretreatment (see Fig. 6A-B). By contrast, higher doses of MitoQ (1–10 μM) exerted better protection when given as pretreatment (see Fig. 6A). In particular, MitoQ (2.5 μM and 5 μM) pretreatment showed significantly higher cell viability (2.19 ± 0.13 and 2.03 ± 0.13) than co-treatment (1.70 ± 0.17 and 1.33 ± 0.20, see Fig. 6). Similarly, SKQ1 also showed better protection at intermediate doses (0.05–1 μM) when given as co-treatment. However, higher doses (5 μM and 10 μM) showed better effects when given as pretreatment. No significant differences were noticed among all the doses of SKQ1 between co-treatment and pretreatment (Fig. 6B).

### Dox significantly increased intracellular ROS and Mitochondrial SO

**Fig. 7:**
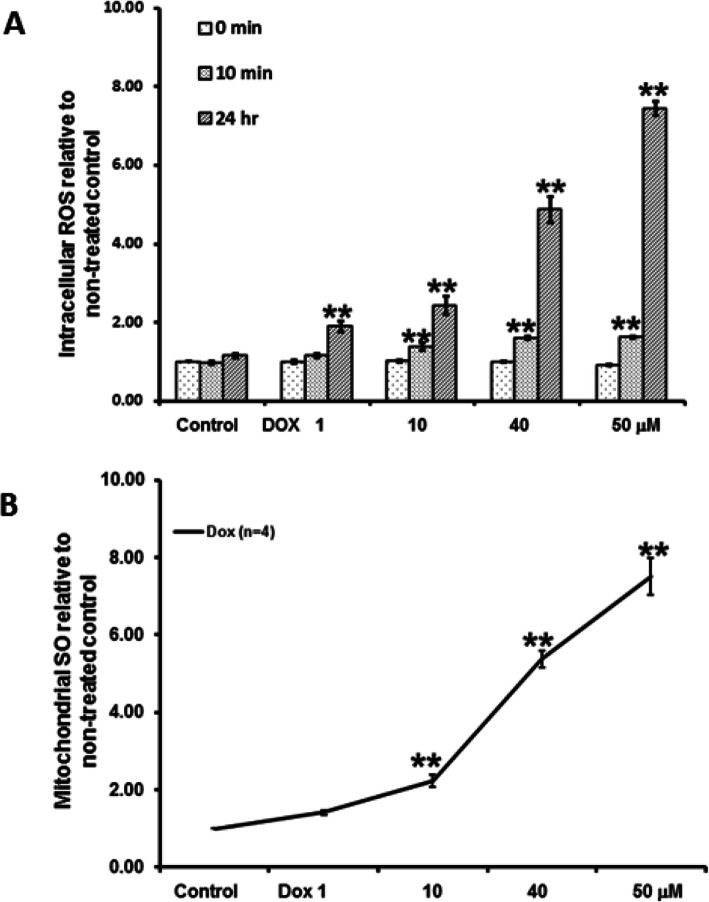
The effects of Dox on intracellular ROS (**A**) and mitochondrial SO (**B**) levels. Dox showed dose-dependent increase in intracellular ROS and mitochondrial SO levels. **P < 0.01 vs the non-treated control

A key mechanism of Dox-induced cardiotoxicity is oxidative stress. The effects of Dox on intracellular ROS and mitochondrial SO are shown in Fig. 7A-B. We found that Dox (1–50 μM, *n* = 3) increased intracellular ROS in a dose and time-dependent manner. We found that the non-treated control only slightly increased intracellular ROS (1.16 ± 0.06) at 24 h when compared to its baseline. Dox (40 μM) significantly increased intracellular ROS at 10 min to 1.61 ± 0.04 fold when compared to the non-treated control baseline. At 24 h, Dox (40 μM) significantly increased intracellular ROS to 4.87 ± 0.34 fold relative to the non-treated control baseline (see Fig. 7A).

Similarly, Dox (1 μM - 50 μM, *n* = 4) increased mitochondrial SO in a dose-dependent manner. At 24 h, Dox (40 μM) significantly increased mitochondrial SO to 5.37 ± 0.21 fold when compared to non-treated control (see Fig. 7B).

### MitoQ and SKQ1 reduced Dox-induced intracellular ROS and mitochondrial SO

**Fig. 8:**
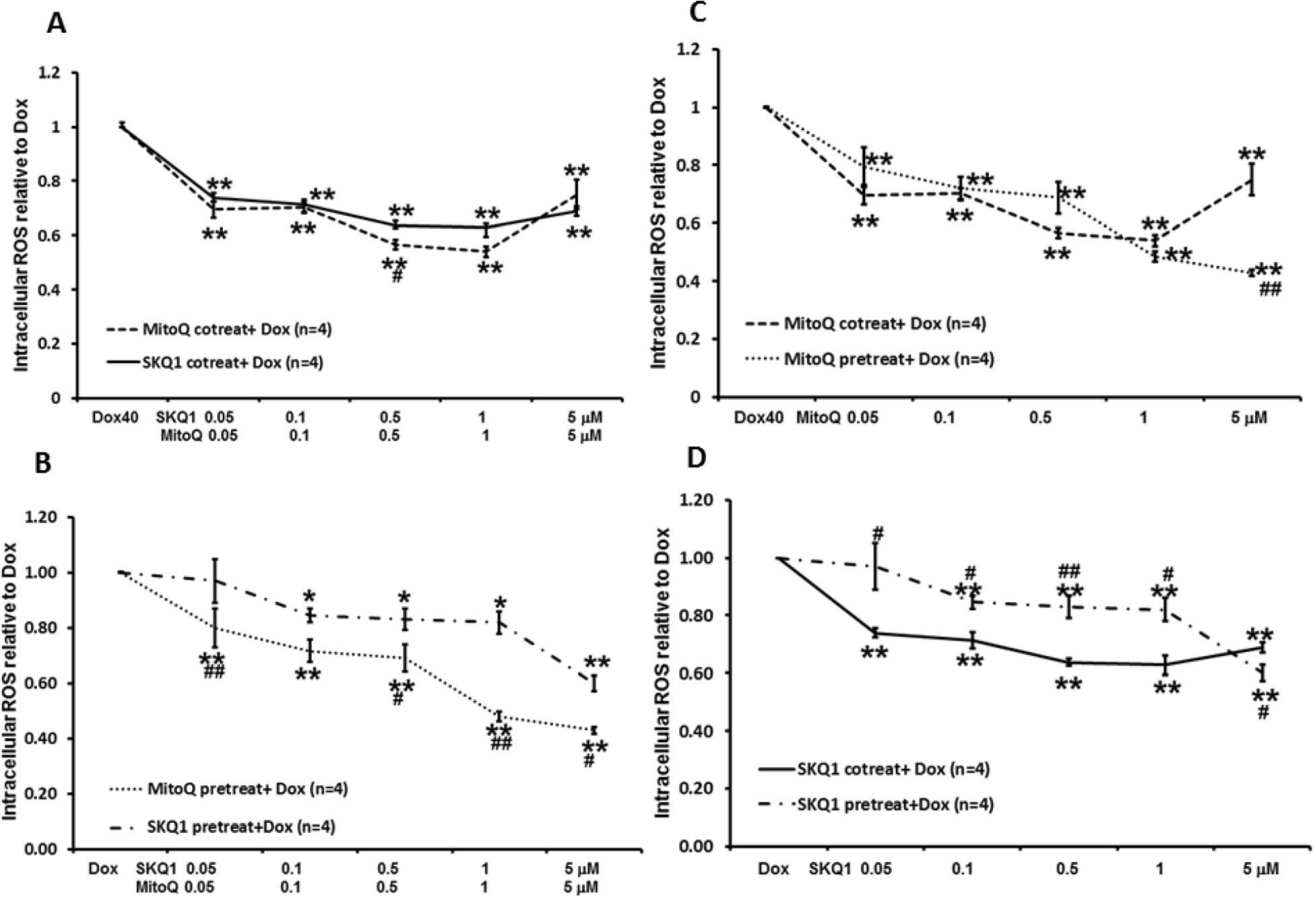
Effects of co-treatment or pretreatment of SKQ1 or MitoQ on Dox-induced increase in intracellular ROS levels. Co-treatment (**A**) and pretreatment (**B**) of SKQ1 and MitoQ significantly attenuated Dox-induced increase in intracellular ROS levels when compared to Dox alone. Pretreatment (**C**-**D**) showed better reduction in intracellular ROS levels than co-treatment only at a higher dose (5 μM). Moreover, MitoQ exerted a significantly higher reduction in intracellular ROS levels than SKQ1. **p < 0.01 vs Dox; #*p* < 0.05, ##*p* < 0.01 vs MitoQ or SKQ1, respectively

The effects of SKQ1 (0.05–5 μM, *n* = 4) and MitoQ (0.05–5 μM, *n* = 4) co-treatment or pretreatment on Dox-induced intracellular ROS levels at 24 h are illustrated in Fig. 8A-D. We found co-treatment or pretreatment of SKQ1 (0.05–5 μM, *n* = 4) and MitoQ (0.05–5 μM, *n* = 4) significantly reduced Dox-induced intracellular ROS levels in a dose-dependent manner (all *p* < 0.05). When given as co-treatment, intermediate dose (1 μM) of SKQ1 and MitoQ showed maximal reduction of intracellular ROS levels to 0.63 ± 0.03 and 0.54 ± 0.02, respectively, when compared to Dox alone (see Fig. 8A). By contrast, when given as pretreatment, higher dose (5 μM) of SKQ1 and MitoQ showed higher reduction of intracellular ROS levels to 0.60 ± 0.03 and 0.43 ± 0.01, respectively, when compared to Dox alone (see Fig. 8B). Both were significantly higher than the corresponding effects of 5 μM of SKQ1 and MitoQ when administered as co-treatment (both *p* < 0.05, see Fig. 8C-D). Moreover, only 0.5 μM MitoQ co-treatment exhibited a significantly higher reduction in intracellular ROS levels when compared to 0.5 μM SKQ1 (*p* < 0.05, see Fig. 8A). By contrast, most doses (0.05, 0.5–5 μM) of MitoQ pretreatment exhibited a significantly higher reduction in intracellular ROS levels than SKQ1 pretreatment (all *p* < 0.05, see Fig. 8B).

**Fig. 9:**
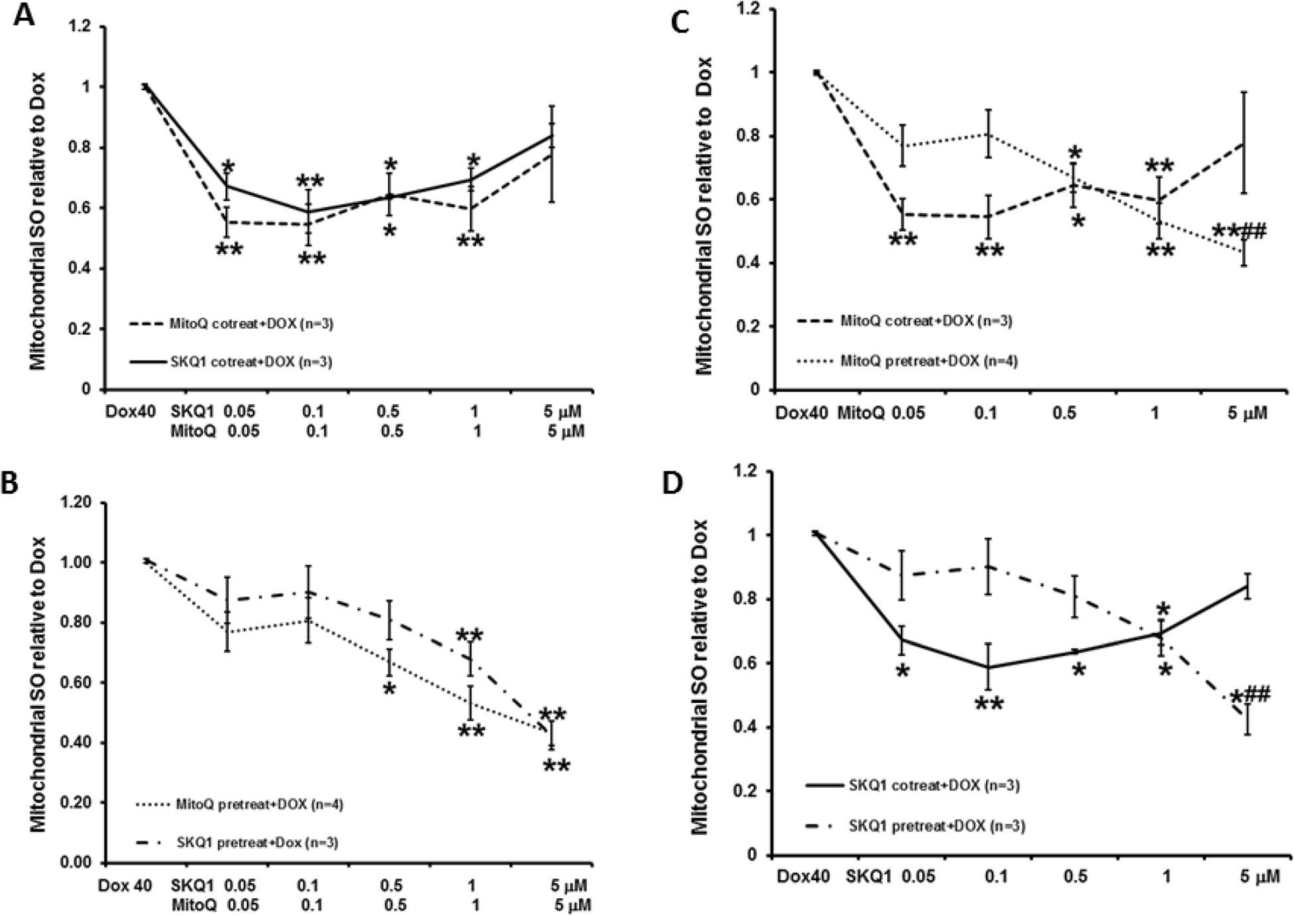
Effects of co-treatment or pretreatment of SKQ1 or MitoQ on Dox-induced increase in mitochondrial SO levels. Co-treatment (**A**) or pretreatment (**B**) of SKQ1 and MitoQ significantly attenuated Dox-induced mitochondrial SO levels. Pretreatment (**C**-**D**) showed better reduction in mitochondrial SO levels than co-treatment only at a higher dose (1–5 μM). Moreover, MitoQ exerted better reduction than SKQ1 at most doses. **p < 0.01 vs Dox; #*p* < 0.05, ##*p* < 0.01 vs MitoQ or SKQ1, respectively

The effects of SKQ1 (0.05–5 μM, *n* = 3) and MitoQ (0.05–5 μM, *n* = 3–4) co-treatment or pretreatment on Dox-induced mitochondrial SO at 24 h are illustrated in Fig. 9A-D. Similarly, co-treatment or pretreatment of SKQ1 and MitoQ significantly reduced mitochondrial SO, although only pretreatment showed a dose-dependent manner (see Fig. 9A-B). Moreover, co-treatment of SKQ1 and MitoQ showed better reduction on mitochondrial SO levels at lower dose range (0.05–1 μM), whereas pretreatment of both drugs showed better effects at higher dose range (1–5 μM). Co-treatment of MitoQ (5 μM) and SKQ1 (5 μM) slightly reduced mitochondrial SO to 0.82 ± 0.04 and 0.82 ± 0.16, respectively, when compared to Dox alone. By contrast, pretreatment of MitoQ (5 μM) and SKQ1 (5 μM) significantly reduced mitochondrial SO to 0.43 ± 0.04 and 0.43 ± 0.05, respectively, when compared to Dox alone (both *p* < 0.01). Both were also significantly higher than the corresponding effects of 5 μM of SKQ1 and MitoQ when administered as co-treatment (both *p* < 0.05, see Fig. 9C-D). Although MitoQ exhibited better reductions in mitochondrial SO at most doses than SKQ1 when given as co-treatment or pretreatment, there were no significant differences.

## Discussion

In summary, we found that Dox (0.5–50 μM) dose-dependently reduced H9c2 cell viability. The effects were similar as those caused by a common oxidative stress inducer, H_2_O_2_. However, unlike H_2_O_2_, there was no difference in Dox cytotoxicity when Dox was added to a fresh medium or 1-day-old medium. Thereafter, we showed that mitochondrial targeted antioxidants, SKQ1or MitoQ, but not the common antioxidant, vitamin C, significantly mitigated Dox-induced cell damage when given as co-treatment or pretreatment. Interestingly, pretreatment of MitoQ provided significantly higher efficacy of cell protection when compared to MitoQ co- treatment or SKQ1 pretreatment. Lastly, we demonstrated that Dox dose-dependently increased intracellular ROS and mitochondrial SO. By contrast, co-treatment or pretreatment of SKQ1 or MitoQ significantly reduced Dox-induced intracellular ROS and mitochondrial SO. However, pretreatment shower higher reduction only at higher doses than co-treatment.

### Dox induced H9c2 cell damage by increasing intracellular and mitochondrial ROS

Dox is a powerful chemotherapeutic drug and widely used in solid cancer treatment [19]. Dox usage is limited due to induction of irreversible cardiotoxicity [1, 20]. This damage is characterized by cardiac cell apoptosis or necrosis which can lead to cardiomyopathy [21]. Our study showed that Dox (0.5–50 μM) dose-dependently decreased H9c2 myoblast cell viability after incubation for 24 h. The cell viability was evaluated by measuring intracellular dehydrogenase activity of viable cells and confirmed by calcein staining. Our results are consistent with a study by Zhang et al. showing that Dox induced cell damage in a dose- and time-dependent manner. Dox (5 μM) caused about 30% reduction in H9c2 cell viability after incubation for 24 h [20], which is similar to our data. By contrast, Dallons et al. showed Dox (1–50 μM) had no effect on cell viability after incubation for 4 h, whereas Dox (1 μM) showed 60% reduction in cell viability after 24 h treatment when cell viability was measured by crystal violet assay [21]. The difference of Dox induced cell damage is possibly due to the sensitivity of methodology used to evaluate cell viability. Moreover, Dallons seeded cells at 3 × 10^4^ cells/cm^2^ and experiments started after cell seeding for 48 h. By contrast, we seeded cells at 6 × 10^4^ cells/cm^2^ and experiments started after cell seeding for 24 h. Researchers have found that cell numbers can influence cell damage effects induced by oxidizer [22].

In this study, we also showed that cell damage induced by H_2_O_2_ could be significantly impacted by fresh medium when compared to 1-day-old medium. The difference may be due to antioxidant effects provided by fetal bovine serum and α-keto acid in fresh medium [23]. However, we did not see any significant difference in cell viability by Dox between fresh medium and 1-day-old medium. Therefore, we could add Dox into the fresh medium after washing out MitQ or SKQ1 after both drugs were given as pretreatment.

Oxidative stress is a major mechanism mediating Dox-induced cardiac damage. It has been found that Dox, a cationic compound, can accumulate in mitochondria 100 times more than in the cytosol due to larger mitochondrial membrane potential (e.g., − 160 to -180 mV). Moreover, Dox interacts with cardiolipin forming electrostatic bonds and disrupting the mitochondrial electron transport chain, particularly complex I and II [24]. In consequence, the dysfunctional electron transport chain facilitates SO generation and overwhelms the mitochondrial antioxidant capacity in the heart. Meanwhile, Dox itself undergoes redox cycling and directly reduces to a semiquinone species through interaction with complex I. This reduced semiquinone version of Dox can oxidize oxygen and form SO. Additionally, Dox can regulate mitochondrial NADPH oxidase increasing ROS production [25]. In this study, we found that Dox increased intracellular ROS in a dose and time-dependent manner. Intracellular ROS measured by DCF fluorescence intensity started to increase after Dox incubation for 10 min and it continued to rise until 24 h. Moreover, we specifically measured mitochondrial SO levels by MitoSOX after Dox incubation for 24 h. We found that the dose-response of Dox-induced mitochondrial SO increase was very similar as the dose-response of Dox-induced intracellular ROS increase at 24 h. The data is consistent with Kuznetsov et al. showing Dox increased intracellular ROS evaluated by measuring DCF fluorescence intensity paralleled with mitochondrial redox state and membrane potential [26]. Asensio-Lopez et al. also showed that Dox dose and time-dependently increased intracellular ROS. Moreover, by utilizing MitoTracker green to locate mitochondria, they found that Dox started to appear in mitochondria at 15 min after administration. It was accompanied with increased ROS fluorescence at mitochondria as well. Both intensities increased as a function of time [25]. In consequence, increased ROS levels in mitochondria lead to membrane potential collapse, ATP production decrease, lipid peroxidation, cytochrome C release and mitochondrial DNA damage [1, 20]. A large amount studies have confirmed that Dox induces apoptosis in cardiomyocytes by increasing cytochrome C and caspase 3 and 9 activity [1, 20, 21].

### Vitamin C failed to protect cells against Dox-induced damage

Because oxidative stress is a major and early event after giving Dox, there are numerous researchers investigating if antioxidants can protect the heart against Dox-induced cardiotoxicity. Most preclinical studies found that common antioxidants, such as vitamin C, can protect the heart against Dox. Viswanatha Swamy et al. showed that vitamin C (20 mg/kg orally) when given 15 days before or 15 days after Dox significantly reduced heart cell damage and oxidative stress by enhancing antioxidant enzymes (e.g., superoxide dismutase, catalase) in rats [27]. Similarly, Akolkar et al. demonstrated better cardiac structure and function in rats when vitamin C (50 mg/kg, orally) was given a week before and continued till 2 weeks after Dox injection. Moreover, they illustrated vitamin C significantly mitigated Dox-induced nitrosative stress, proapoptotic proteins (e.g., caspase 3), inflammatory cytokines (e.g., interleukin 6), and the relevant signaling proteins (e.g., p53, nuclear factor kappa light chain enhancer of activated B cells, autophagy) in rats and isolated cardiomyocytes [5, 8, 28]. However, it is still a lack of sufficient clinical research evidence to prove the effectiveness of vitamin C in the attenuation of cardiotoxicity caused by Dox [29, 30].

In this study, we expected that vitamin C would mitigate Dox-induced cell damage. Moreover, it could serve as an effective control to compare mitochondrial-targeted antioxidants’ effects. We first found that vitamin C (1–2000 μM) alone only slightly increased cell viability after incubation for 24 h. However, when vitamin C (1–2000 μM) was applied concurrently with Dox, vitamin C failed to show any protective effects. Moreover, higher doses of vitamin C (1000 μM and 2000 μM) showed slightly lower cell viability than Dox alone. So far, vitamin C has not shown any protection against Dox-induced cardiotoxicity in clinical studies [29, 30]. This may be because vitamin C is not able to reach sources of ROS (e.g. mitochondria) quickly enough to scavenge ROS or its potential pro-oxidant property. Vitamin C is hydrophilic and it gets into the cell or cellular organelles by Na-dependent vitamin C transporter or glucose transporter. AKolkar et al. showed that Dox downregulated the expression of both transporter proteins in the cardiomyocytes [8]. Moreover, the distributions of vitamin C in different intracellular compartments vary in different tissues. For example, vitamin C concentration is five times less in the mitochondria of mouse skeletal muscle than that in the liver [31]. Furthermore, studies indicate that vitamin C can become pro-oxidant at higher concentration (e.g., > 1000 μM), higher intracellular transition metal ions, or dysfunctional mitochondria [32–34]. In addition, a higher Dox concentration (e.g., 40 μM) was used in this study than other studies (e.g., 10 μM). All the above factors may contribute to why vitamin C showed no cell protection against Dox in this study.

### MitoQ and SKQ1, given as pretreatment, mitigated Dox-induced cell damage at higher degree than co-treatment

Due to Dox mainly accumulating in the mitochondria to induce oxidative stress, mitochondrial-targeted antioxidants would be more efficient to mitigate Dox-induced cell damage. MitoQ and SKQ1 are two well-studied mitochondrial-targeted antioxidants [35]. They are ubiquinone and plastoquinone, respectively, conjugated to a TPP, a lipophilic cation. The conjugated drugs accumulate several hundred times more in the mitochondria than cytosol due to larger membrane potential within the cell [10, 36]. Inside mitochondria, MitoQ and SKQ1 switch between their reduced and oxidized form via the electron transport chain, with the reduced form able to scavenge ROS [15, 37]. However, MitoQ and SKQ1 can be pro-oxidants at higher doses [15]. We found that MitoQ (0.05–5 μM) and SKQ1 (0.05–10 μM) alone slightly increased cell viability when compared to non-treated control. By contrast, a higher dose of MitoQ (10 μM) slightly reduced H9c2 cell viability by 5%. Mendez et al. also showed that MitoQ (10 μM) is cytotoxic to platelets [38].

It is well-known that heart preconditioning by transient ischemia/reperfusion episodes allows the heart develop resilience to endure a harsher insult, such as prolonged ischemia/reperfusion injury [39]. Currently, this strategy has been tried to mitigate Dox-induced cardiotoxicity. Maulik et al. showed that simulated preconditioning by hypoxia/reoxygenation attenuated Dox-induced cell damage in primary adult cardiac myocytes. However, a ROS scavenger, N-acetyl cysteine, failed to show any protection when given concurrently with Dox [16]. Similarly, Galan-Arriola et al. found that remote ischemia preconditioning before intracoronary injection of Dox preserved significantly better left ventricular ejection fraction, mitochondrial morphology, and DNA copies in pigs’ heart [17]. Instead of using ischemic preconditioning, we pretreated cells with mitochondrial targeted antioxidants, MitoQ or SKQ1, 24 h prior to Dox in this study. We also compared the effects of pretreatment to those of co-treatment. We found both co-treatment and pretreatment mitigated Dox-induced cell damage in H9c2 myoblast cells. However, the dose-responses were different between co-treatment and pretreatment. Co-treatment showed significant protection at intermediate doses of MitoQ (0.5–1 μM) and SKQ1 (1 μM); whereas higher doses showed reduced protection. By contrast, when given as pretreatment, higher doses of MitoQ (1–10 μM) and SKQ1 (5 μM) were required to protect the cells against Dox. Moreover, the efficacy of protection by MitoQ pretreatment was significantly better than its co-treatment. We further found that the protection may be related to the dose-dependent reduction in intracellular ROS and mitochondrial SO. It is noticeable that both drug’s antioxidant effects shared the similar shift of dose-dependent response between co-treatment and pretreatment. To our knowledge, the present study showed that pretreatment of mitochondrial-targeted antioxidants has higher efficacy against Dox-induced cell damage than co-treatment for the first time. The difference possibly is due to the following reasons: 1. Accumulation of MitoQ or SKQ1 in mitochondria depends on mitochondrial membrane potential [10]. When the drugs were co-administered with Dox, Dox dissipated the mitochondrial membrane potential which possibly reduced the drug accumulation in mitochondria; 2. It has been reported that higher doses of MitoQ and SKQ1 can be pro­oxidants. In particular, MitoQ shows higher pro-oxidant property at lower doses than SKQ1 in vitro studies [13]. Moreover, Huang et al. found that MitoQ (10 μM) reduced mitochondrial membrane potential in pancreatic acinar cells [40]. We found that higher doses of SKQ1 or MitoQ (e.g. 5 and 10 μM) when given as co-treatment showed less cell protection or no protection at all. 3. MitoQ/SKQ1 and Dox exert effects via the electron transport chain and cardiolipin, which may lead to interference when given at the same time. For example, Dox needs to be converted to semiquinone causing ROS increase by complex I. By contrast, MitoQ relies on complex I and II to recycle between the reduced and oxidized forms [41]. Similarly, SKQ1 recharges itself between reduced and oxidized forms via complex II and complex III [37]. Furthermore, Dox and SKQ1 showed higher affinity for mitochondrial cardiolipin than MitoQ. However, Dox binding disrupts cardiolipin whereas SKQ1or MitoQ protects cardiolipin from oxidation to preserve mitochondria’s normal function [24, 42]. By contrast, when MitoQ or SKQ1 was applied before Dox, drug accumulation into mitochondria and the antioxidant capacity was unlikely influenced. All the above presumptions warrant further investigation to support that pretreatment would be a better strategy when giving mitochondrial-targeted antioxidants.

Additionally, we demonstrated that MitoQ exhibited significantly higher efficacy against Dox than SKQ1 when both were given as pretreatment. The cellular protection may be partially related to its higher reduction on intracellular ROS and mitochondrial SO than SKQ1 as our data suggested. Plenty of studies support the concept that MitoQ can mitigate Dox-induced cardiotoxicity by reducing oxidative stress [1, 13]. By contrast, SKQ1 has not been widely studied in Dox-induced cardiotoxicity. However, it has been used in eye drops as a defense against oxidative stress due to dry eye syndrome [43]. SKQ1 has been also studied to promote survival of kidney epithelial cells and significantly improved survival of rats subjected to ischemia/reperfusion injury by reducing oxidative stress [44]. In vitro studies suggest that SKQ1 has antioxidant effects at lower concentrations than MitoQ [13]. Moreover, the reduced form of SKQ1 has a four-fold higher decrease in peroxyl radicals than the reduced form of MitoQ [11]. In addition to their antioxidant ability, Hu et al. recently indicated that MitoQ pretreatment activated Nrf2 signaling to enhance antioxidant capacity and to protect mitochondrial DNA in an intestinal ischemia/reperfusion model [18]. The role of Nrf2 signaling in cardioprotection provided by pretreatment of MitoQ or SKQ1 needs to be further elucidated.

### Limitation

We acknowledge that this study was performed on a rat H9c2 cardiomyoblast cell line instead of primary cultured cardiomyocytes. However, Kuznetsov et al. compared H9c2 cells’ mitochondrial biogenesis, function and response to hypoxia/reoxygenation to primary cardiomyocytes’. They suggested that H9c2 cells were very similar to primary heart cells regarding the energy metabolism and mitochondrial properties [45]. Therefore, we would like to further validate the effects of pretreatment of mitochondrial-targeted antioxidants in a Dox-induced cardiotoxicity animal model. Moreover, we did not evaluate whether Dox’s anti-cancer effects would be compromised by using mitochondrial-targeted antioxidants as co-treatment or pretreatment. Rao et al. demonstrated that MitoQ exerted 30 times more cytotoxicity to breast cancer cell lines than to healthy mammary epithelial cells [46]. Moreover, they also found that MitoQ not only increased Dox’s anti-cancer effects but also mitigated Dox-induced cardiotoxicity [47]. Similarly, SKQ1 also showed to attenuate cell growth in fibrosarcoma and rhabdomyosarcoma tumor cell lines and related animal models [48]. However, given the possibility of competition when mitochondrial-targeted antioxidants are co-treated with Dox, it will be very intriguing to find out if administration of MitoQ or SKQ1 as pretreatment prior to Dox would provide better anti-cancer effects than co-treatment. It will also shine some light on how cancer patients can safely take antioxidants and not interfere with anti-cancer treatments’ benefits.

## Conclusion

This present study showed that Dox dose-dependently reduced cell viability of H9c2 cells by increasing intracellular ROS and mitochondrial SO. Mitochondrial targeted antioxidants, MitoQ and SKQl, but not vitamin C, significantly mitigated Dox-induced cell damage when given as co-treatment or pretreatment. Interestingly, MitoQ pretreatment showed significantly higher efficacy in cellular protection than MitoQ co-treatment and SKQ1 pretreatment. The protective effects by MitoQ and SKQ1 were associated with significant reduction in intracellular ROS and mitochondrial SO. The data also demonstrate that pretreating cardiomyocytes by MitoQ prior to Dox may exert significantly better cardioprotection than co-treatment. The mechanisms underlying the difference between co-treatment and pretreatment against Dox-induced cardiac cell damage and the impacts on Dox’s anti-cancer effects will be explored in future studies.

## Data Availability

The datasets generated during and/or analyzed during the current study are available from the corresponding author on reasonable request.

## Abbreviations

Dox: Doxorubicin
TOP II: Topoisomerase II
ROS: Reactive oxygen species
MitoQ: Mitoquinone
SKQ1: 10-(6′-plastoquinonyl) decyltriphenylphosphonium
TPP: Decyl-triphenylphosphonium
ATCC: American Type Culture Collection
H_2_O_2_: Hydrogen peroxide
CCK-8: Cell counting kit-8
DCFDA: 2, 7-dichlorofluorescin diacetate
SO: Superoxide
